# Supplementary material for: An Orthogonal Experimental Study on the Preparation of Cr Coatings on Long-Size Zr Alloy Tubes by Arc Ion Plating
Source: Materials (Basel). 2022 Oct 14;15(20):7177. doi: 10.3390/ma15207177 (PMC9611484; doi:10.3390/ma15207177)
Supplement: Supplementary file 1 [file materials-15-07177-s001.zip › materials-1847782-supplementary.pdf]

materials

Supplementary materials

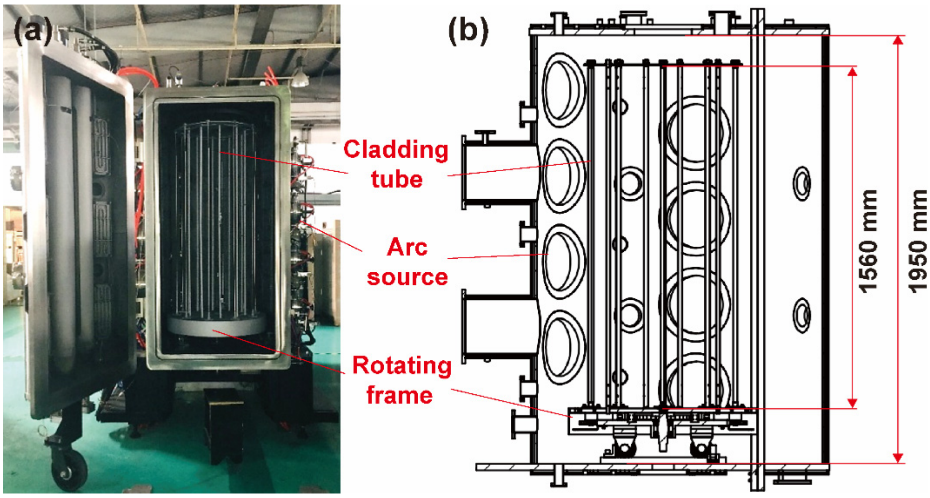

Figure S1. Industrial multi-arc ion plating equipment with a vertical side-door configuration: (a) overall appearance and (b) side perspective view.

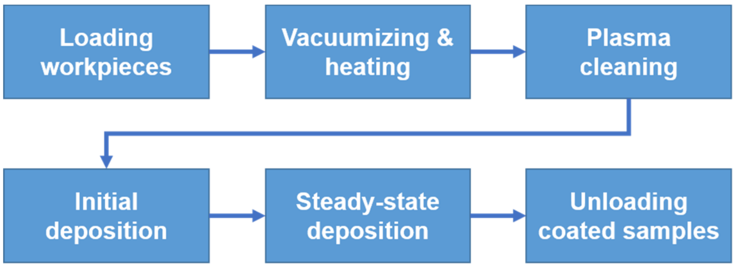

Figure S2. Flow-process diagram of coating fabrication.

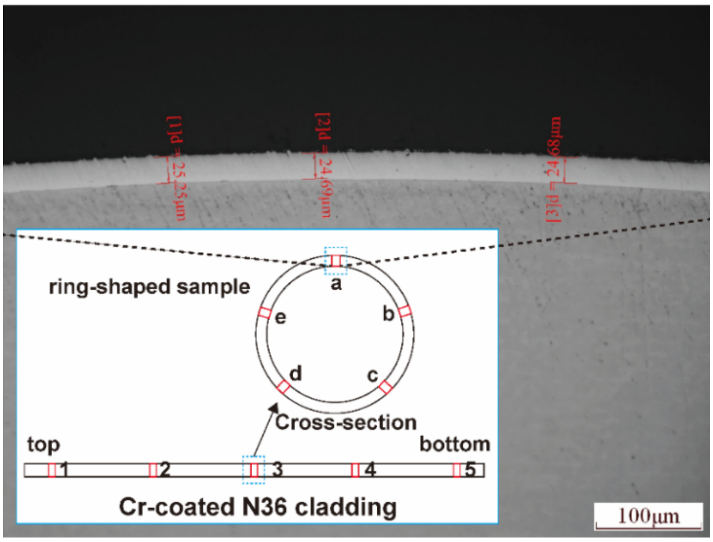

Figure S3. Cross-section optical microscopy image of Cr-coated N36 cladding tube. The lower-left inset shows the sampling diagram of the tube in axial and circumferential directions.

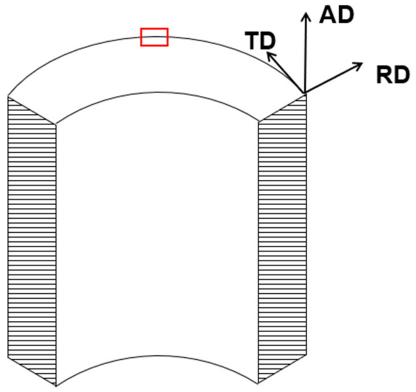

Figure S4. Coordinate diagram of EBSD samples.

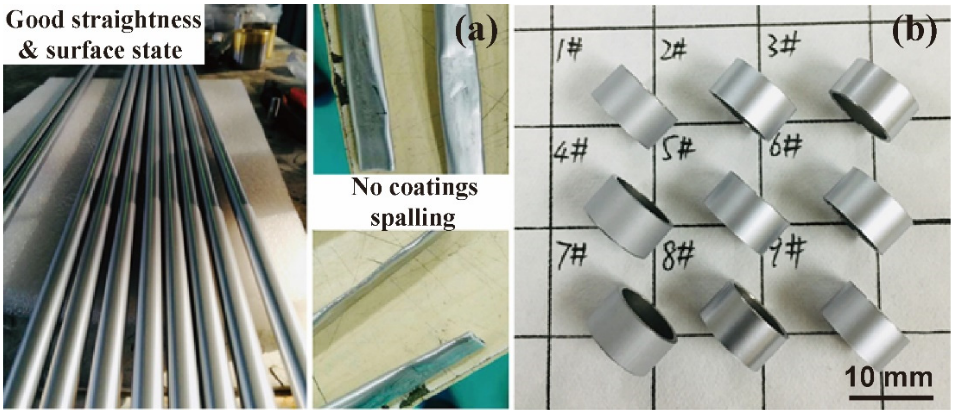

Figure S5. Macro-photos of as-prepared Cr-coated N36 tube samples: (a) overall appearance and on-site destructive inspection; (b) appearance of the orthogonal samples obtained under nine different process conditions.

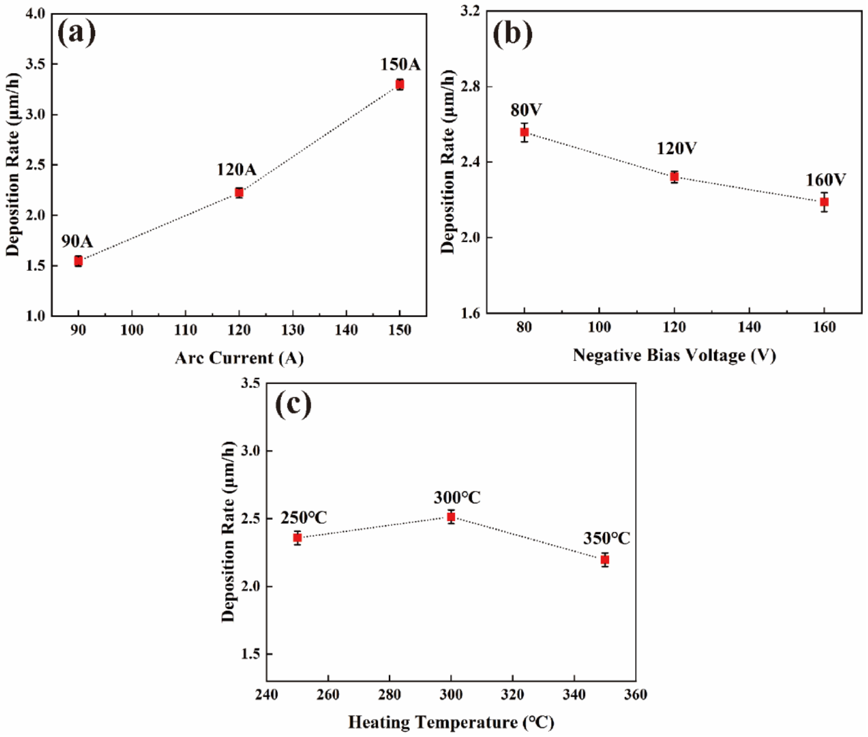

Figure S6. Average deposition rate of Cr coating as a function of (a) arc current, (b) negative bias voltage and (c) heating temperature, respectively.

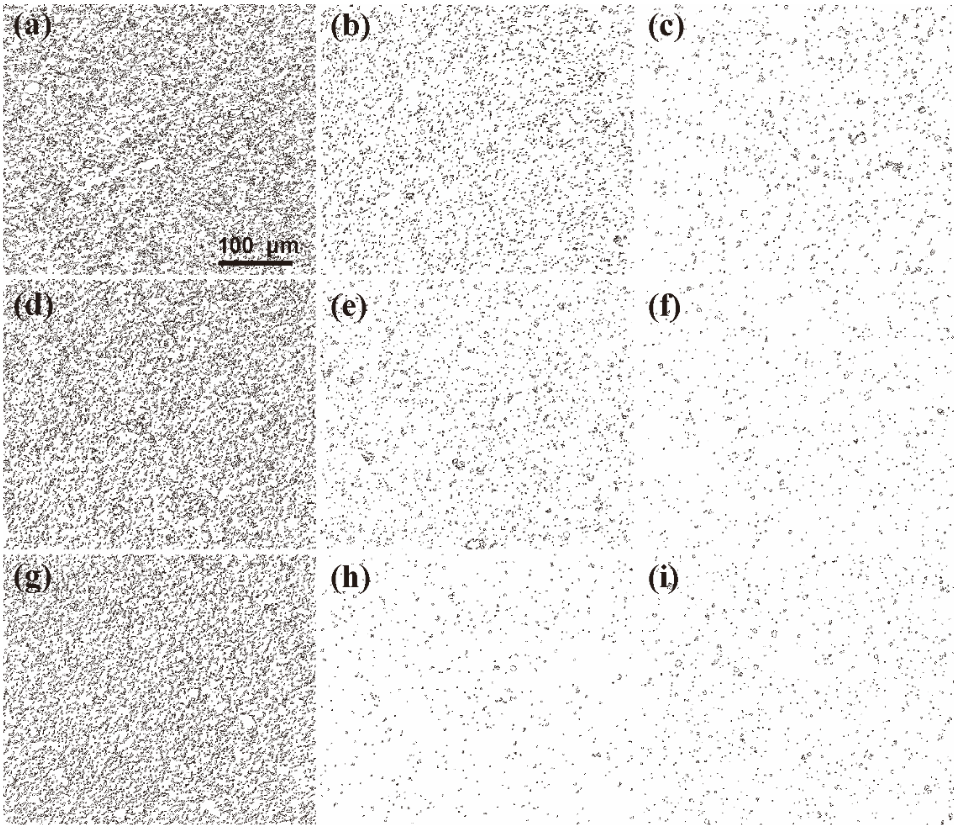

Figure S7. Profiles of particles on the surface of Cr coatings obtained via Image J software: (a-i) corresponding to #1-9 samples respectively. All images are of the same scale.

Table S1. Results of the range and variance calculations of surface roughness (Ra) and particle numbers (Count).

Index	Factor level	Temperature (°C)	Arc current (A)	Gas pressure (Pa)	Bias voltage (V)
Ra	k_{1j}	0.64	0.78	0.58	0.65
	k_{2j}	0.62	0.54	0.62	0.56
	k_{3j}	0.55	0.49	0.61	0.59
	R_j	0.09	0.30	0.04	0.09
	SS_j	0.0131	0.1501	0.0033	0.0126
Count	k_{1j}	2943	4551	2017	2577
	k_{2j}	2434	1911	2799	2750
	k_{3j}	1993	909	2555	2043
	R_j	950	3642	782	707
	SS_j	1355090	21235585	961873	814348

Table S2. F-testing results of surface roughness (Ra) and particle numbers (Count).

Variance source	SS_j	df_j	MS_j	F	Table value	Significance level
**Ra**						
Arc current	0.1501	2	0.0750	45.94	$F_{0.022}(2,2)=44.45$	α ~ 0.022
Bias voltage	0.0126	2	0.0063	3.86	$F_{0.26}(2,2)=3.85$	α ~ 0.206
Temperature	0.0131	2	0.0065	4	$F_{0.2}(2,2)=4$	α ~ 0.2
Error	0.0033	2	0.0016			
Sum	0.1791	8				
**Count**						
Arc current	21235585	2	10617792	20.35	$F_{0.003}(2,6)=17.80$	α ~ 0.003
Temperature$^{\triangle}$	1355090	2	677545			
Gas pressure$^{\triangle}$	961873	2	480936			
Error	814348	2	407174			
Error$^{\triangle}$	3131310	6	521885			
Sum	24366895	8				

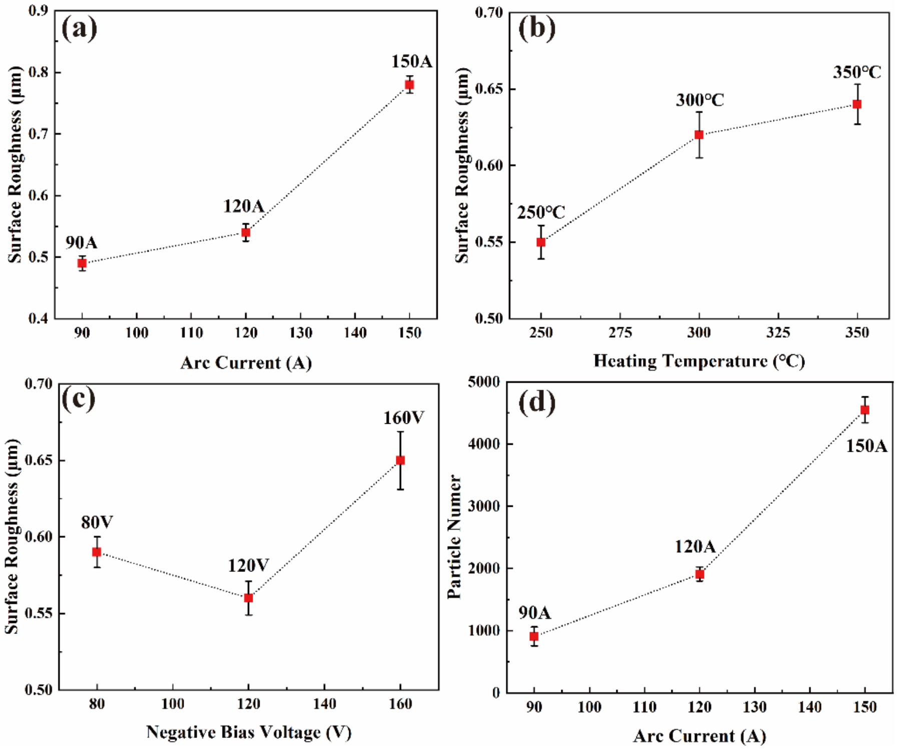

Figure S8. Surface roughness of Cr coating as a function of (a) arc current, (b) heating temperature and (c) negative bias voltage, respectively, and (d) number of particles as a function of arc current.

Table S3. Results of the range and variance calculations of TC_{200} and TC_{211}.

Index	Factor level	Temperature (°C)	Arc current (A)	Gas pressure (Pa)	Bias voltage (V)
TC_{200}	k_{1j}	0.656	0.573	0.575	0.819
	k_{2j}	0.663	0.696	0.739	0.619
	k_{3j}	0.737	0.787	0.742	0.618
	R_j	0.081	0.214	0.167	0.201
	SS_j	0.012086	0.069206	0.054794	0.080402
TC_{211}	k_{1j}	0.653	0.656	0.513	**0.734**
	k_{2j}	0.611	**0.725**	0.552	0.533
	k_{3j}	0.662	0.545	**0.861**	0.659
	R_j	0.051	0.180	0.348	0.201
	SS_j	0.004446	0.049482	0.218106	0.061902

Table S4. F-testing results of TC_{200} and TC_{211}.

Variance source	SS_j	df_j	MS_j	F	Table value	Significance level
			TC_{200}			
Arc current	0.069206	2	0.034603	5.73	$F_{0.149}(2,2)=5.71$	α ~ 0.149
Gas pressure	0.054794	2	0.027397	4.53	$F_{0.181}(2,2)=4.52$	α ~ 0.181
Bias voltage	0.080402	2	0.040201	6.65	$F_{0.131}(2,2)=6.63$	α ~ 0.131
Error	0.012086	2	0.006043			
Sum	0.216488	8				
			TC_{211}			
Arc current	0.049482	2	0.024741	11.13	$F_{0.083}(2,2)=11.05$	α ~ 0.083
Gas pressure	0.218106	2	0.109053	49.06	$F_{0.02}(2,2)=49.00$	α ~ 0.02
Bias voltage	0.061902	2	0.030951	13.92	$F_{0.068}(2,2)=13.71$	α ~ 0.068
Error	0.004446	2	0.002223			
Sum	0.333936	8				

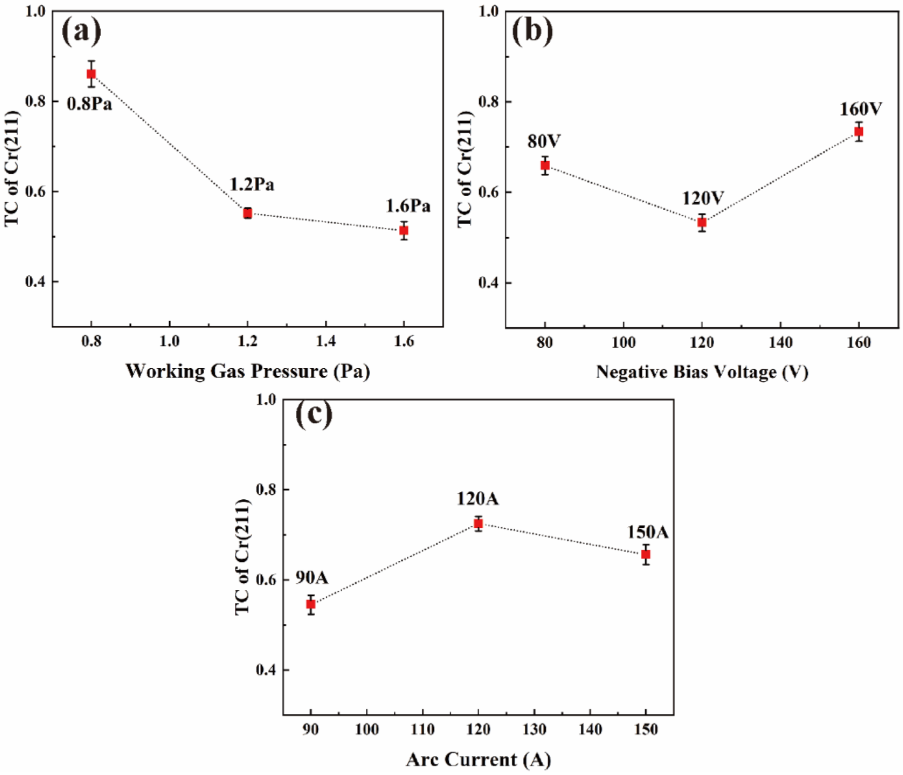

Figure S9. TC_{211} of Cr coatings as functions of (a) gas pressure, (b) negative bias voltage and (c) arc current, respectively.

Table S5. Range and variance calculation results of average diameter ($\bar{d}$) and aspect ratio ($\overline{L/W}$) of Cr grains.

Index	Factor level	Temperature (°C)	Arc current (A)	Gas pressure (Pa)	Bias voltage (V)
$\bar{d}$	k_{1j}	1.623	1.615	1.767	1.452
	k_{2j}	1.710	1.851	1.533	1.697
	k_{3j}	1.576	1.443	1.610	1.760
	R_j	0.134	0.408	0.234	0.308
	SS_j	0.027829	0.251273	0.085334	0.159349
$\overline{L/W}$	k_{1j}	4.008	3.666	3.903	3.510
	k_{2j}	4.169	4.165	3.909	4.056
	k_{3j}	3.609	3.955	3.973	4.220
	R_j	0.560	0.499	0.070	0.710
	SS_j	0.498401	0.376045	0.009032	0.828785

Table S6. F-testing results of average diameter ($\bar{d}$) and aspect ratio ($\overline{L/W}$) of Cr grains.

Variance source	SS_j	df_j	MS_j	F	Table value	Significance level
			$\bar{d}$			
Arc current	0.251273	2	0.125636	9.03	$F_{0.1}(2,2)=9.00$	α ~ 0.1
Bias voltage	0.159349	2	0.079674	5.73	$F_{0.149}(2,2)=5.71$	α ~ 0.149
Gas pressure	0.085334	2	0.042667	3.07	$F_{0.25}(2,2)=4.52$	α ~ 0.25
Error	0.027829	2	0.013914			
Sum	0.523784	8				
			$\overline{L/W}$			
Bias voltage	0.828785	2	0.414392	91.76	$F_{0.011}(2,2)=89.91$	α ~ 0.011
Temperature	0.498401	2	0.249200	55.18	$F_{0.018}(2,2)=54.56$	α ~ 0.018
Arc current	0.376045	2	0.188022	41.63	$F_{0.024}(2,2)=40.67$	α ~ 0.024
Error	0.009032	2	0.004516			
Sum	1.712262	8				

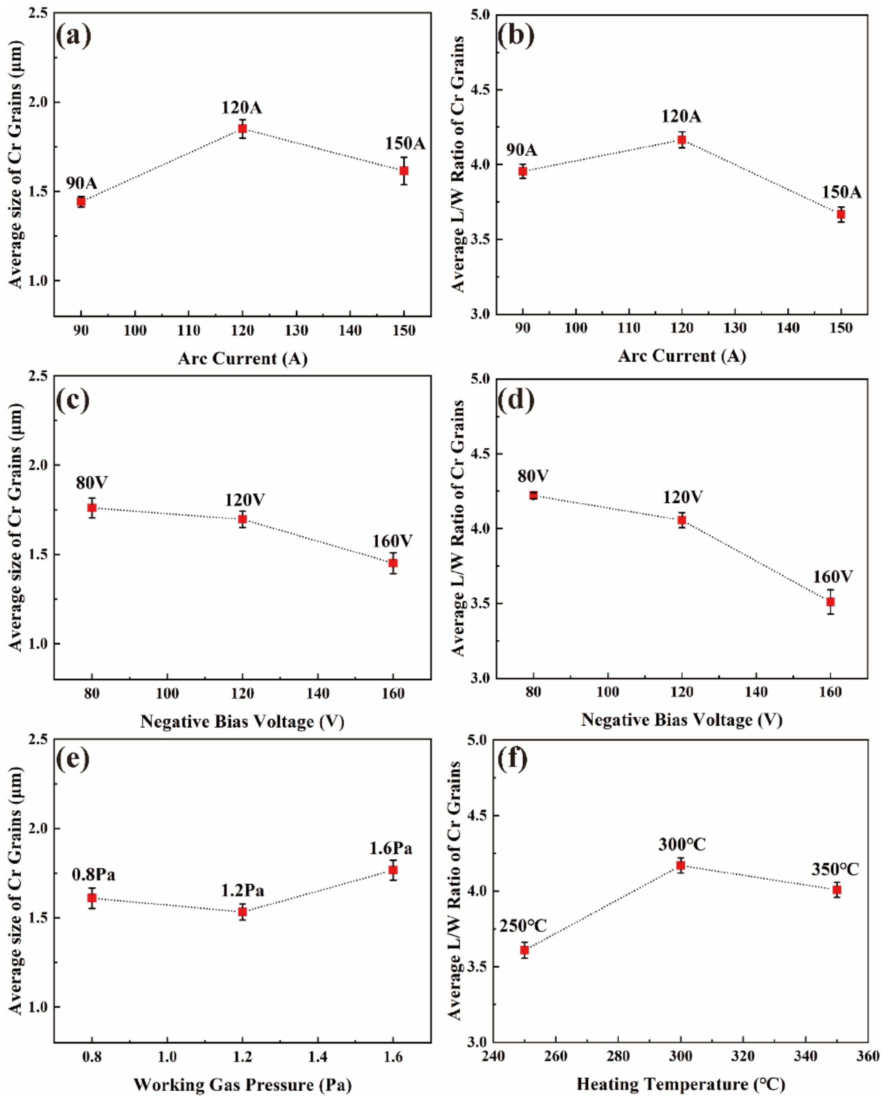

Figure S10. Average size (a, c, e) and aspect ratio (b, d, f) of Cr grains as functions of various process parameters.
